# The Association Between Body Mass Index, Physical Fitness and COVID-19 Hospitalization Among Male Active Duty U.S. Army Soldiers, May 2020–November 2021

**Published:** 2025-10-01

**Authors:** Jacob D. Smith, Joseph R. Pierce, Anthony Marquez, Ryan Steelman, Markku A. Malmi, Michelle Canham-Chervak, John F. Ambrose

**Affiliations:** Preventive Medicine Division, Defense Centers for Public Health–Aberdeen, Aberdeen Proving Ground, MD: Mr. Smith, Mr. Marquez, Dr. Ambrose; Military Injury Prevention Division, Defense Centers for Public Health–Aberdeen: Dr. Pierce, Dr. Canham-Chervak

## Abstract

Few studies have investigated body mass index (BMI) and physical fitness factors related to coronavirus disease (COVID)-19 hospitalizations among U.S. active duty service members. This investigation examined associations between measures of physical fitness, BMI, and Army physical fitness test (APFT) performance with COVID-19 hospitalizations of U.S. Army active duty soldiers. From May 2020 through November 2021, 13,074 male soldiers were diagnosed with COVID-19 (90 hospitalized, 12,984 non-hospitalized) who also had an APFT and BMI record no more than 9 months from the COVID-19 diagnosis date. Female soldiers were excluded due to insufficient numbers of COVID-19 hospitalizations. In adjusted logistic regression models controlling for race and ethnicity as well as comorbidities, and including age, BMI, and their interactions, both BMI (adjusted odds ratio [aOR] 1.07; 95% CI 1.01, 1.14; *p* =0.021), and the age and BMI interaction were statistically significant (aOR 1.01; 95% CI 1.00, 1.02; *p* =0.004). Each additional year of age amplified the odds of hospitalization by an additional 1% for every 1 unit increase in BMI. Development and maintenance of a healthy body weight may reduce likelihood of COVID-19 hospitalization and sustain individual and unit health and medical readiness.

 Although the U.S. Centers for Disease Control and Prevention (CDC) has identified well-established risk factors—such as age, sex, race, comorbidities, vaccination status—for coronavirus disease (COVID)-19 hospitalization within the general U.S. population, limited research has explored the contributing factors specific to U.S. active duty service members. ^1 , 2^

 Obesity (BMI > 30 kg/m ^2^ ) is perhaps the most common comorbidity associated with COVID-19 severity, but obesity is related to several other chronic conditions including hypertension, type 2 diabetes, cardiovascular disease, lung disease, and sleep apnea, all of which have been independently associated with severe COVID-19 disease. ^3 - 7^ Additionally, overweight (BMI 25.0–29.9 kg/m ^2^ ) or obesity increase risk of respiratory symptoms, such as shortness of breath, often associated with severe COVID-19 outcomes. ^4 , 6 , 8^ Service members are estimated to have higher overweight prevalence and lower obesity prevalence compared to the general U.S. population, with similar trends of higher overweight prevalence with older age. ^9^

 A 2021 CDC *Morbidity and Mortality Weekly Report* added further evidence that a higher BMI increases risk of severe COVID-19 outcomes (e.g., hospitalization, intensive care unit hospitalization, or death) in the general public. ^4^ Epsi et al. (2021) reported that obesity was correlated with COVID-19 severity in a study of Military Health System (MHS) beneficiaries, in which active duty service members comprised over 50% of the study population. ^3^ Early in the pandemic, studies described comorbidities associated with positive COVID-19 cases in the U.S. Army active duty population, and included obesity diagnosis codes in the medical records. Studies have yet to examine associations with BMI values obtained from periodic body composition assessments, such as the Army's Digital Training Management System (DTMS) or vital records associated with medical encounters. ^1 , 2^

**What are the new findings?**

For male U.S. Army active duty soldiers, the association between having a higher BMI and COVID-19 hospitalization was amplified by age, indicating about a 1% increase in the odds of hospitalization per BMI unit for each additional year of age.

**What is the impact on readiness and force health protection?**

Maintaining a healthy body weight may reduce the risk of COVID-19 related hospitalization for military personnel. The U.S. Army's Holistic Health and Fitness Program is one example of a comprehensive health program established to simultaneously enhance several facets of military health and fitness.

The active duty military population tends to be more physically fit, younger, and healthier (i.e., ‘the healthy soldier effect’ or ‘healthy worker effect’) compared to the general U.S. population due to accession requirements for health, ready access to medical care, and stringent standards of physical fitness and body composition. ^10 - 12^ The current U.S. Army *Field Manual* , volume 7-22, *Holistic Health and Fitness* , describes the Holistic Health and Fitness (H2F) Program that prescribes physical readiness training at least 5 to 6 times per week for a total of 5 to 7.5 hours in addition to rigorous fitness standards. ^13^

 Physical activity is 1 of 4 main modifiable risk factors identified by the CDC to reduce risk of some chronic diseases, which have been associated with severe COVID-19 outcomes. ^6 , 14^ Regular physical activity is generally associated with improved immune response, reduction in comorbid conditions, and reduction in systemic inflammation. ^15 , 16^ Regular physical activity has also been shown to reduce susceptibility to viral infection; however, this is dependent on meeting guidelines for exercise volume and intensity. ^17^ Greater cardiorespiratory fitness may provide improved pro-inflammatory responses and increased antiviral host responses post-infection. ^15 , 16^ A meta-analysis of almost 2 million medical records demonstrated a reduction in risk of COVID-19 infection, hospitalization, and mortality for individuals who participated in regular physical activity (e.g., 500 meta-bolic equivalent [MET]-minutes per week, where 1 MET equals resting energy expenditure and MET-minutes is the product of METs achieved and task duration) compared to individuals who were inactive (0 MET-minutes per week). ^18^

 While prior studies have compared pre- and post-pandemic impacts on physical activity and BMI, few studies have described how physical fitness and BMI, prior to COVID-19 diagnosis, affected COVID-19 hospitalizations. ^18 - 21^ One large retrospective study in 2020 found that physically inactive patients diagnosed with COVID-19 were significantly more likely to experience severe COVID-19 outcomes including hospitalization, intensive care unit (ICU) admission, or death. ^21^ This report describes associations between prior BMI and prior physical fitness performance with COVID-19 hospitalization while adjusting for age, race and ethnicity, vaccination status, and comorbidities. 

## Methods

### Study population

**FIGURE 1.**
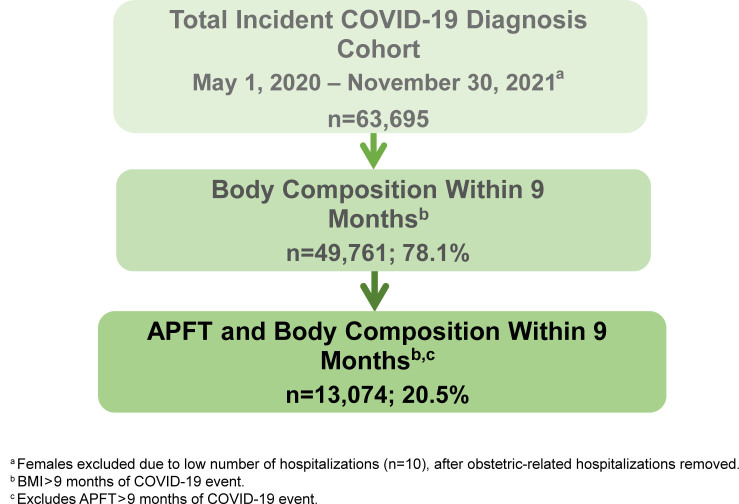
Analysis Population Exclusions, Male Active Duty U.S. Army Soldiers with Incident COVID-19 Diagnosis, BMI and APFT, May 2020–November 2021

 The population for this retrospective cohort study included U.S. Army active duty soldiers with measured heights and weights and either 1) documented history of initial COVID-19 or 2) history of initial COVID-19 hospitalization from May 1, 2020 through November 30, 2021. (See **Figure 1** for analysis population exclusions.) The beginning of the period was selected to capture the widespread use of the ICD-10-CM (International Classification of Diseases, 10th Revision, Clinical Modification) U07.1 diagnosis code for COVID-19. The end of the period was selected to capture cases before the initial wave of the Omicron variant, in December 2021. 

Administrative medical data were obtained in December 2022 from electronic health records in the Military Health System Data Repository (MDR), and reportable medical event data were obtained from the Disease Reporting System internet (DRSi). The MDR is one of the most robust centralized sources of Department of Defense (DOD) health care data. MDR data utilized for this report included inpatient and outpatient medical encounters, immunizations, laboratory results, and pharmacy records.

 COVID-19 hospitalizations were included if the first 2 positions of the diagnostic codes in the inpatient medical records contained 1 of the COVID-19 ICD-10-CM diagnosis codes **(Table 1)** and occurred within 30 days of the initial COVID-19 diagnosis or positive SARS-CoV-2 (severe acute respiratory syndrome coronavirus 2) polymerase chain reaction (PCR) laboratory result or DRSi medical event report. ^2 , 22 - 25^ Non-hospitalized COVID-19 encounters were defined by a COVID-19 ICD-10-CM diagnosis code **(Table 1)** in the first 2 diagnostic positions, a positive SARS-CoV-2 PCR laboratory result, or a confirmed DRSi case without a related inpatient record. 

**TABLE 1.** ICD-10-CM Diagnosis Codes Utilized to Identify COVID-19 Hospitalizations

Description	ICD-10-CM
Coronavirus, unspecified	B34.2
SARS-associated coronavirus as the cause of disease classified elsewhere	B97.21
Other coronavirus as the cause of diseases classified elsewhere	B97.29
Acute nasopharyngitis; common cold	J00
Acute upper respiratory infection; unspecified	J06.9
Pneumonia due to SARS-associated coronavirus	J12.81
Pneumonia due to coronavirus disease 2019	J12.82
Other viral pneumonia	J12.89
Viral pneumonia unspecified	J12.9
Pneumonia due to other specified infectious organism	J16.8
Pneumonia in diseases classified elsewhere	J17
Bronchopneumonia, unspecific organism	J18.0
Lobar pneumonia, unspecified organism	J18.1
Other pneumonia, unspecified organism	J18.8
Pneumonia, unspecified organism	J18.9
Acute bronchitis due to other specified organisms	J20.8
Acute bronchitis, unspecified	J20.9
Unspecified acute lower respiratory infection	J22
Bronchitis, not specified as acute or chronic	J40
Acute respiratory distress syndrome	J80
Idiopathic interstitial pneumonia not otherwise specified	J84.111
Acute respiratory failure	J96.0
Cough	R05
Dyspnea	R06.0
Dyspnea, unspecified	R06.00
Shortness of breath	R06.02
Acute respiratory distress	R06.03
Other forms of dyspnea	R06.09
Anosmia	R43.0
Aguesia	R43.2
Fever, unspecified	R50.9
2019-nCoV acute respiratory disease, COVID-19, virus identified	U07.1

Abbreviations: ICD-10-CM, International Classification of Diseases, 10th Revision, Clinical Modification; COVID-19, coronavirus disease 2019; SARS, severe acute respiratory syndrome; 2019-nCoV, 2019 novel coronavirus.

Vaccination status at the date of COVID-19 diagnosis was obtained from MDR immunization, outpatient, and pharmacy data using ‘CVX’, ‘CPT’, and ‘NDC’ codes. Soldiers completing a primary COVID-19 vaccination series were defined as those who had received the second dose of a 2-dose primary vaccination series or a single dose of a 1-dose primary vaccine product 14 days or more prior to a COVID-19 encounter. Soldiers with 1 dose of a 2-dose primary vaccination series were categorized as ‘partially vaccinated’, and others were categorized as ‘unvaccinated’.

 A soldier was considered to have a comorbidity if a medical encounter contained an ICD-10-CM diagnosis code for that condition in any diagnosis position from January 1, 2019 and the date of the initial positive COVID-19 diagnosis. Comorbidities were selected using Clinical Classifications Software Refined (CCSR) categories from diagnostic codes similar to other research by the CDC, with a retrospective review period through January 1, 2019. ^4 , 26 , 27^ CCSR categories used included hypertension (CIR007, CIR008), coronary atherosclerosis and other heart disease (CIR011), chronic kidney disease (GEN003), diabetes (END002, END003), neoplasms (CIR categories beginning with ‘NEO’), chronic obstructive pulmonary disease and bronchiectasis (RSP008), and sleep wake disorders (NVS016). ^4 , 26 , 27^

 Active duty soldier demographics (i.e., service, component, age, sex, race and ethnicity) were obtained in December 2022 from Defense Manpower Data Center (DMDC) personnel rosters. Age was calculated at the COVID-19 encounter date by date of birth. Race and ethnicity were categorized, based on data available in DMDC, as 1) non-Hispanic White—the reference population—2) non-Hispanic Black, 3) Hispanic, or 4) ‘other’ including those of Asian, Native Hawaiian/Pacific Islander, American Indian / Alaskan Native, or other race or ethnicity. BMI (displayed as kg/m ^2^ ) was calculated using height (inches) and weight (pounds) closest to the initial COVID-19 encounter date using the formula (weight [lb] / height [in] ^2^ ) x 703). Measurements were recorded during periodic height and weight checks by unit personnel in Defense Training Management System (DTMS) body composition records, supplemented by MDR vital records recorded during medical encounters when no DTMS record was available. Records were included if the BMI measurement was no more than 9 months prior to the documented COVID-19 diagnosis date. 

DTMS data for the Army physical fitness test (APFT) were used because those data were more readily available during the investigation period; the Army combat fitness test (ACFT) was not yet the U.S. Army fitness test of record. The APFT assessed physical fitness through performance on 3 timed events: 1) 2-minute push-ups, 2) 2-minute sit-ups, and 3) a 2-mile run. APFT event data were retained if the record occurred no more than 9 months prior to the initial COVID-19 diagnosis date, were considered ‘for record’, and each of the 3 events contained plausible values recorded (e.g., push-ups and sit-ups of 1-150 repetitions, 2-mile run times of 9.5–30 minutes). Implausible values accounted for less than 0.1% of all records.

### Exclusions

Records were excluded if a soldier had a history of COVID-19 prior to the investigation start date, as identified via DRSi or the medical record, or were non-active duty (including activated National Guard or reserve). Female service members were excluded from the analysis due to an insufficient number (n=10) of hospitalizations after obstetric-related admissions were removed.

### Statistical analysis

Differences in COVID-19 hospitalization by categorical variables were explored with chi-square tests; continuous variables were explored using univariate logistic regression. Crude and adjusted logistic regression models were fit to estimate odds ratios (ORs) and associated 95% confidence intervals (CIs). Adjusted logistic regression models used the outcome of COVID-19 hospitalization and age and BMI as main predictors, controlling for covariates that included race and ethnicity, vaccination status, comorbidities, and physical fitness characteristics. An interaction term between age and BMI was also included in the model.

 Non-linearity was assessed using empirical logistic plots and the functional form with cumulative residual plots. When non-linearity was detected, models were fit as a linear term, polynomial degree, and restricted cubic splines, and the fit (i.e., AIC) of the linear term with the non-linear term was compared. Initial covariate selection was *a priori* , considering both linear and non-linear terms for each variable, as appropriate. Variables were excluded if the non-linear term did not improve the model fit compared to the linear term. Variables with less than 15 observations per category were excluded. There was strong evidence of non-linearity among the 3 APFT variables. Even after fitting different models with various functional forms of the 3 APFT variables, the model fit did not improve, and the APFT variables were omitted from the adjusted model. The final adjusted models included racial and ethnic group, age, BMI, comorbidities, and an interaction between age and BMI. Alpha levels were set to 0.05. Analyses were conducted using SAS, version 9.4 (SAS Institute Inc., Cary, NC). 

## Results

 From May 1, 2020 through November 30, 2021, a total of 13,074 unique male Army active duty soldiers were identified as incident COVID-19 cases with a documented BMI and complete 3-event APFT no more than 9 months prior to the COVID-19 encounter date **(Figure 1)** . Women were excluded from the analysis because only 10 hospitalizations of female soldiers for COVID-19 occurred, which was below the minimum required for analysis. 

**Table 2** summarizes the baseline demographic, physical fitness, and body composition characteristics of this cohort. The average male soldier was 26.5 years old (standard deviation [SD] 6.0) with a BMI of 26.6 (SD 3.4). Those male soldiers performed an average of 63.6 push-ups (SD 12.9), 67.3 sit-ups (SD 10.9), and completed the 2-mile-run in 14.9 minutes (SD 1.5) on the APFT **(Table 2)** . The cohort was primarily non-Hispanic White (51.4%), unvaccinated (95.9%), with no histories of the selected comorbidities (91.4%) **(Table 2)** . Compared with soldiers who were hospitalized, those not hospitalized were younger, with lower BMI, performed more sit-ups, and had a lower proportion of comorbidities **(Table 2)** . Only 3% of soldiers were fully vaccinated during the study period, and just 4 of those were hospitalized; consequently, vaccination status was not incorporated in the adjusted model. 

**TABLE 2.** Characteristics of COVID-19-Hospitalized Versus Non-Hospitalized Male Active Duty U.S. Soldiers, May 2020–November 2021

COVID-19-related Outcome				
	Total	Hospitalized	Non-Hospitalized	*p* -value
Total, *n*	13,074	90	12,984	
Continuous Variables				
Age, *y*				0.004
Mean ± SD	26.5 ± 6.0	28.3 ± 7.1	26.5 ± 6.0	
Median (IQR)	25.0 (22.0, 29.0)	27.0 (22.0, 34.0)	25.0 (22.0, 29.0)	
BMI (kg/m ^2^ )				<0.001
Mean ± SD	26.6 ± 3.4	27.9 ± 4.0	26.6 ± 3.4	
Median (IQR)	26.3 (24.3, 28.7)	28.0 (25.0, 30.9)	26.3 (24.3, 28.7)	
APFT push-ups (repetitions)				0.189
Mean ± SD	63.6 ± 12.9	61.8 ± 12.2	63.6 ± 12.9	
Median (IQR)	65.0 (54.0, 74.0)	62.5 (55.0, 71.0)	65.0 (54.0, 74.0)	
APFT sit-ups (repetitions)				0.003
Mean ± SD	67.3 ± 10.9	63.9 ± 9.8	67.3 ± 10.9	
Median (IQR)	67.0 (60.0, 76.0)	64.0 (57.0, 71.0)	67.0 (60.0, 76.0)	
APFT 2-mile run (minutes)				0.060
Mean ± SD	14.9 ± 1.5	15.2 ± 1.3	14.9 ± 1.5	
Median (IQR)	14.8 (14.0, 15.7)	15.4 (14.3, 16.1)	14.8 (13.9, 15.7)	
Categorical Variables				

	No.	%	No.	%	No.	%	
Race and ethnicity							0.066
White, non-Hispanic	6,714	51.4	41	45.6	6,673	51.4	
Black, non-Hispanic	2,826	21.6	27	30.0	2,799	21.6	
Hispanic	2,707	20.7	13	14.4	2,694	20.7	
Other	827	6.3	9	10.0	818	6.3	
Vaccination status							0.981
Unvaccinated	12,541	95.9	86	95.6	12,455	95.9	
Partial	124	0.9	1	1.1	123	0.9	
Full	409	3.1	3	3.3	406	3.1	
Comorbidities							0.006
No history	11,952	91.4	75	83.3	11,877	91.5	
History	1,122	8.6	15	16.7	1,107	8.5	

Abbreviations: *n* , number; *y* , years; SD, standard deviation; IQR, interquartile range; No., number; kg, kilogram; m, meter; APFT, Army physical fitness test.

 In unadjusted analyses, BMI (OR 1.11; 95% CI 1.05, 1.17), age (OR 1.04; 95% CI 1.01, 1.08), sit-ups (OR 0.97; 95% CI 0.95, 0.99), and comorbidities (OR 2.15; 95% CI 1.23, 3.75) were each significantly associated with COVID-19-related hospitalization **(Table 3)** . 

**TABLE 3.** Unadjusted Association Between BMI, APFT and COVID-19 Hospitalization, Male Active Duty U.S. Army Soldiers, May 2020–November 2021

	No.	OR	95% CI Lower Limit	95% CI Upper Limit	*p* -value
Total	13,074				
Continuous variables ^a^					
BMI (kg/m ^2^ )	13,074	1.11	1.05	1.17	<0.001
Age, *y*	13,074	1.04	1.01	1.08	0.004
APFT push-ups, *n*	13,074	0.99	0.97	1.01	0.189
APFT sit-ups, *n*	13,074	0.97	0.95	0.99	0.003
APFT 2-mile run (min)	13,074	1.12	1.00	1.26	0.060
Race and ethnicity					
White, non-Hispanic	6,714	Reference	—	—	—
Black, non-Hispanic	2,826	1.57	0.96	2.56	0.070
Hispanic	2,707	0.79	0.42	1.47	0.449
Other	827	1.79	0.87	3.7	0.115
Comorbidities					
History	1,122	2.15	1.23	3.75	<0.001
No history	11,952	Reference	—	—	—
Vaccination status					
Unvaccinated	12,541	Reference	—	—	—
Partial	124	1.18	0.16	8.52	0.871
Full	409	1.07	0.34	3.4	0.908

Abbreviations: BMI, body mass index; APFT, Army physical fitness test; COVID-19, coronavirus disease 2019; No., number; OR, odds ratio; CI, confidence interval; kg, kilogram; m, meter; *y* , years; *n* , number; min, minute.

a Continuous variables were modeled per 1 unit increase unless otherwise specified.

**FIGURE 2.**
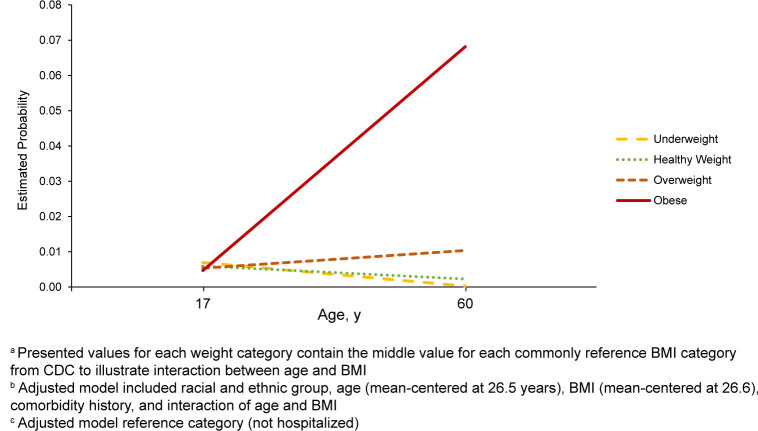
BMI and Age Interaction-Adjusted Probabilities for COVID-19 Hospitalization, Male Active Duty U.S. Army Soldiers, May 2020–November 2021

 The final adjusted model included race and ethnicity, age, BMI, comorbidities, and the interaction term for age (mean-centered at 26.5 years old) and BMI (mean-centered at 26.6 kg/m ^2^ ). In the adjusted model, the main effect of age was not statistically significant (aOR 1.01; 95% CI 0.98, 1.05), whereas the main effect of BMI was significant, with an additional 7% increase in the adjusted odds (aOR 1.07; 95% CI 1.01, 1.14) **(Table 4)** . The age and BMI interaction was significant, for each additional year of age, the adjusted odds with a 1-unit increase in BMI is amplified by an additional 1%, and conversely each additional BMI unit amplifies the age effect by an additional 1% **(Table 4** , **Figure 2)** . 

**TABLE 4.** Adjusted Association Between BMI and COVID-19 Hospitalization, Male Active Duty U.S. Army Soldiers, May 2020–November 2021

	No.	aOR	95% CI Lower Limit	95% CI Upper Limit	*p* -value
Total	13,074				
Continuous variables ^a^					
Age ^b^ , *y*	13,074	1.01	0.98	1.05	0.451
BMI ^b^ (kg/m ^2^ )	13,074	1.07	1.01	1.14	0.021
BMI x age ^b^	13,074	1.01	1.00	1.02	0.004
Race and ethnicity					
White, non-Hispanic	6,714	Reference	—	—	—
Black, non-Hispanic	2,826	1.50	0.92	2.45	0.108
Hispanic	2,707	0.73	0.39	1.37	0.330
Other	827	1.63	0.79	3.4	0.187
Comorbidities					
History	1,122	1.32	0.69	2.5	0.401
No history	11,952	Reference	—	—	—

Abbreviations: BMI, body mass index; COVID-19, coronavirus disease 2019; No., number; aOR, adjusted odds ratio; CI, confidence interval; *y* , years; kg, kilogram; m, meter.

a Continuous variables were modeled per 1 unit increase unless otherwise specified.

b BMI x age results are mean-centered (mean BMI 26.6, mean age 26.5).

## Discussion

This study investigated the association between BMI, physical fitness, and COVID-19 hospitalizations in a subset of U.S. Army active duty soldiers with an APFT and body composition measures no more than 9 months prior to a COVID-19 medical encounter, either hospitalized or non-hospitalized. Prior physical fitness, as measured by APFT performance, in this cohort was not associated with COVID-19 hospitalization. In the adjusted logistic regression model, at the average age, each 1 unit increase in BMI increased odds of hospitalization by 7%. Additionally, there was significant interaction between BMI and age, with an additional 1% increase in odds of hospitalization for each unit increase in either BMI or age.

 The lack of association between prior physical fitness and COVID-19 hospitalization found in this study is inconsistent with some studies which suggested that higher levels of prior physical fitness could lessen likelihood of COVID-19 hospitalization. ^18 , 28 - 30^ Differences in the methods that defined and measured physical fitness, along with the study populations, complicate direct comparisons between these results and those prior reports. Other papers have evaluated self-reported physical fitness or self-reported physical activity, which may introduce self-reporting and recall bias. ^21 , 29^ One report evaluating maximal exercise capacity, via peak METs, used fitness tests up to 2 years prior to SARS-CoV-2 infection and included a population unrepresentative of the U.S. population with a significantly higher hospitalization rate compared to other reports. ^30^

 At least 1 study of U.S. service members identified self-reported fitness and exercise capacity decrements following SARS-CoV-2 infection. ^31^ A specific threshold of physical fitness could potentially reduce hospitalization duration or intensity. Alternatively, physical fitness may reduce symptom duration or intensity during a non-hospitalized infection, which this report did not assess. This could also be due to the multifactorial nature of COVID-19 severity, in which other factors such as pre-existing health conditions, age, immune response, and genetic predispositions play critical roles. Additionally, the ‘healthy warrior effect’, attributed to rigorous physical and medical screening processes required for military service, health care access, and employment, may also positively affect clinical outcomes. ^10^ Active duty soldiers who are generally healthier and more physically fit may experience lower morbidity, which could have influenced this study's observed associations. Soldiers participate in regular physical activity to maintain required physical fitness standards, and several studies and a meta-analysis found that regular physical activity was associated with lower risk of COVID-19 infection, hospitalization, severe illness, and death. ^18 - 21^

 The significant interaction found in this study between BMI and age underscores the compounded risk that higher BMI and increasing age pose for hospitalization. This finding aligns with existing literature that has identified obesity as a major risk factor for hospitalization, likely due to the association and interaction of COVID-19 with comorbidities such as hypertension, diabetes, and cardiovascular diseases. ^2 , 4 , 32 , 33^ Other reports that examined changes in service member BMI during the same period observed a significant increase in obesity, although the increases tended to be largest among service members younger than age 20 years. ^34^ The additional 1% increase in hospitalization risk per unit increase in BMI with age in this study suggests that some older individuals with higher BMI are particularly vulnerable, highlighting the need for targeted interventions in this group. This report differed from other studies that primarily relied on an ICD-10-CM diagnosis code to indicate obesity rather than measured heights and weights to calculate BMI. ^1 , 2^ This approach enabled us to better understand the relationship between BMI, age, and COVID-19-related hospitalization observed in our models. 

 This study has several limitations. Soldiers with a BMI and APFT record no more than 9 months from the COVID-19 diagnosis date limited the sample size to 20.5% of the original population, which could affect the generalizability of the results **(Figure 1)** . The sample size available for soldiers with an APFT was considerably lower during this period, primarily due to fitness testing pauses during the initial stages of the COVID-19 pandemic (i.e., “lockdowns”). As the pandemic continued, the ACFT was gradually phased in, until established as the fitness test of record on October 1, 2022, resulting in fewer available APFT results. The ACFT data were incomplete and unavailable for use during the reporting period. It is also possible that ACFT performance may demonstrate different associations with COVID-19 hospitalizations than the APFT, given that it assesses additional physical fitness components (e.g., anaerobic fitness, muscular strength and power); ACFT results were not widely available during the period investigated, however. Because soldiers are automatically enrolled in TRICARE, the number of cases and related characteristics may have been under-estimated if soldiers sought care outside of the MHS TRICARE network or were unreported in DRSi. Vaccination status may have been under-estimated due to the accessibility of vaccinations at out-of-network facilities, such as pharmacies or mass vaccination sites. 

COVID-19 hospitalizations may not be entirely preventable, but the results of this analysis suggest that risk is higher among military personnel with higher BMI and greater age. Resources available to soldiers such as H2F and Armed Forces Wellness Centers can provide individual guidance to maintain or improve BMI.

## References

[1] Kebisek J , Forrest LJ , Maule AL , Steelman RA , Ambrose JF . Prevalence of selected underlying health conditions among active component Army service members with coronavirus disease 2019, 11 February–6 April 2020 . MSMR . 2020 ; 27 ( 5 ): 50 - 54 . Accessed Sep. 15, 2025 . https://www.health.mil/reference-center/reports/2020/05/01/medical-surveillance-monthly-report-volume-27-number-5 32479103

[2] Stidham RA , Stahlman S , Salzar TL . Cases of coronavirus disease 2019 and comorbidities among Military Health System beneficiaries, 1 January 2020 through 30 September 2020 . MSMR . 2020 ; 27 ( 12 ): 2 - 8 . Accessed Sep. 15, 2025 . https://www.health.mil/reference-center/reports/2020/12/01/medical-surveillance-monthly-report-volume-27-number-12 33393791

[3] Epsi NJ , Richard SA , Laing ED , et al . Clinical, immunological, and virological SARS-CoV-2 phenotypes in obese and nonobese military health system beneficiaries . J Infect Dis . 2021 ; 224 ( 9 ): 1462 - 1472 . doi: 10.1093/infdis/jiab396 34331541 PMC8385847

[4] Kompaniyets L , Goodman AB , Belay B , et al . Body mass index and risk for COVID-19–related hospitalization, intensive care unit admission, invasive mechanical ventilation, and death—United States, March–December 2020 . MMWR Morb Mortal Wkly Rep . 2021 ; 70 ( 10 ): 355 - 361 . doi: 10.15585/mmwr.mm7010e4 33705371 PMC7951819

[5] Rebello CJ , Kirwan JP , Greenway FL . Obesity, the most common comorbidity in SARS-CoV-2: is leptin the link? Int J Obes (Lond) . 2020 ; 44 ( 9 ): 1810 - 1817 . doi: 10.1038/s41366-020-0640-5 32647360 PMC7347260

[6] U.S. Centers for Disease Control and Prevention . People with Certain Medical Conditions. U.S. Dept. of Health and Human Services . Accessed Sep. 9, 2021 . https://www.cdc.gov/coronavirus/2019-ncov/need-extra-precautions/people-with-medical-conditions.html

[7] Ng R , Sutradhar R , Yao Z , Wodchis WP , Rosella LC . Smoking, drinking, diet and physical activity: modifiable lifestyle risk factors and their associations with age to first chronic disease . Int J Epidemiol . 2020 ; 49 ( 1 ): 113 - 130 . doi: 10.1093/ije/dyz078 31329872 PMC7124486

[8] Salome CM , King GG , Berend N . Physiology of obesity and effects on lung function . J Appl Physiol (1985) . 2010 ; 108 ( 1 ): 206 - 211 . doi: 10.1152/jap-plphysiol.00694.2009 19875713

[9] Eilerman PA , Herzog CM , Luce BK , et al . A comparison of obesity prevalence: Military Health System and United States populations, 2009–2012 . Mil Med . 2014 ; 179 ( 5 ): 462 - 470 . doi: 10.7205/milmed-d-13-00430 24806489

[10] McLaughlin R , Nielsen L , Waller M . An evaluation of the effect of military service on mortality: quantifying the healthy soldier effect . Ann Epidemiol . 2008 ; 18 ( 12 ): 928 - 936 . doi: 10.1016/j.annepidem.2008.09.002 19041592

[11] Under Secretary of Defense for Personnel and Readiness . Department of Defense Instruction Number 1304.26. Qualification Standards for Enlistment, Appointment, and Induction . U.S. Dept. of Defense . Updated May 29, 2025 . Accessed Sep. 15, 2025 . https://www.esd.whs.mil/portals/54/documents/dd/issuances/dodi/130426p.pdf

[12] Chowdhury R , Shah D , Payal AR . Healthy worker effect phenomenon: revisited with emphasis on statistical methods: a review . Indian J Occup Environ Med . 2017 ; 21 ( 1 ): 2 - 8 . doi: 10.4103/ijoem.ijoem_53_16 29391741 PMC5763838

[13] Headquarters, Department of the Army . Field Manual 7-22: Holistic Health and Fitness . Change 2. U.S. Dept. of Defense . Updated Aug. 2025 . Accessed Sep. 15, 2025 . https://armypubs.army.mil/epubs/dr_pubs/dr_a/arn44522-fm_7-22-002-web-7.pdf

[14] Ng R , Sutradhar R , Yao Z , Wodchis WP , Rosella LC . Smoking, drinking, diet and physical activity-modifiable lifestyle risk factors and their associations with age to first chronic disease . Int J Epidemiol . 2020 ; 49 ( 1 ): 113 - 130 . doi: 10.1093/ije/dyz078 31329872 PMC7124486

[15] Burtscher J , Millet GP , Burtscher M . Low cardiorespiratory and mitochondrial fitness as risk factors in viral infections: implications for COVID-19 . Br J Sports Med . 2021 : 55 ( 8 ) 413 - 415 . doi: 10.1136/bjsports-2020-103572 33234508

[16] Da Silveira MP , da Silva Fagundes KK , Bizuti MR , et al . Physical exercise as a tool to help the immune system against COVID-19: an integrative review of the current literature . Clin Exp Med . 2021 ; 21 ( 1 ): 15 - 28 . doi: 10.1007/s10238-020-00650-3 32728975 PMC7387807

[17] Piercy KL , Troiano RP , Ballard RM , et al . The physical activity guidelines for Americans . JAMA . 2018 ; 320 ( 19 ): 2020 - 2028 . doi: 10.1001/jama.2018.14854 30418471 PMC9582631

[18] Ezzatvar Y , Ramírez-Vélez R , Izquierdo M , Garcia-Hermoso A . Physical activity and risk of infection, severity and mortality of COVID-19: a systematic review and non-linear dose–response meta-analysis of data from 1 853 610 adults . Br J Sports Med . 2022 ; 56 ( 20 ): 1188 - 1193 . doi: 10.1136/bjsports-2022-105733 35995587

[19] Cho DH , Lee SJ , Jae SY , et al . Physical activity and the risk of COVID-19 infection and mortality: a nationwide population-based case-control study . J Clin Med . 2021 ; 10 ( 7 ): 1539 . doi: 10.3390/jcm10071539 33917558 PMC8038831

[20] Lee SW , Lee J , Moon SY , et al . Physical activity and the risk of SARS-CoV-2 infection, severe COVID-19 illness and COVID-19 related mortality in South Korea: a nationwide cohort study . Br J Sports Med . 2022 ; 56 ( 16 ): 901 - 912 . doi: 10.1136/bjsports-2021-104203 34301715

[21] Sallis R , Young DR , Tartof SY , et al . Physical inactivity is associated with a higher risk for severe COVID-19 outcomes: a study in 48 440 adult patients . Br J Sports Med . 2021 ; 55 ( 19 ): 1099 - 1105 . doi: 10.1136/bjsports-2021-104080 33849909

[22] U.S. Centers for Disease Control and Prevention . ICD-10-CM Official Coding Guidelines: Supplement: Coding Encounters Related to COVID-19 Coronavirus Outbreak . U.S. Dept. of Health and Human Services . Accessed Mar. 4, 2022 . https://www.cdc.gov/nchs/data/icd/ICD-10-CM-Official-Coding-Gudance-Interim-Advice-coronavirus-feb-20-2020.pdf

[23] National Center for Health Statistics, U.S. Centers for Disease Control and Prevention . ICD-10-CM Official Guidelines for Coding and Reporting. U.S. Dept. of Health and Human Services . Accessed Mar. 4, 2022 . https://www.cdc.gov/nchs/data/icd/ICD-10cmguidelines-FY2021-COVID-up-date-January-2021-508.pdf

[24] National Center for Health Statistics, U.S. Centers for Disease Control and Prevention . ICD-10-CM Official Guidelines for Coding and Reporting FY 2021–UPDATED January 1, 2021 . U.S. Dept. of Health and Human Services . Accessed Sep. 9, 2021 . https://www.cdc.gov/nchs/data/icd/ICD-10cmguidelines-FY2021-COVID-update-January-2021-508.pdf

[25] Armed Forces Health Surveillance Division . Armed Forces Reportable Medical Events Guidelines and Case Definitions . Defense Health Agency, U.S. Dept. of Defense . Accessed Aug. 14, 2023 . https://www.health.mil/reference-center/publications/2022/11/01/armed-forces-reportable-medical-events-guidelines

[26] Healthcare Cost & Utilization Project User Support . Chronic Condition Indicator (CCI) for ICD-10-CM. Agency for Healthcare Research and Quality . Updated Jul. 2025 . Accessed Sep. 15, 2025 . https://www.hcup-us.ahrq.gov/toolssoftware/chronic_icd10/chronic_icd10.jsp

[27] Healthcare Cost & Utilization Project User Support . Clinical Classifications Software Refined (CCSR) . Agency for Healthcare Research and Quality . Updated Nov. 2024 . Accessed Sep. 15, 2025 . https://hcup-us.ahrq.gov/toolssoftware/ccsr/ccs_refined.jsp

[28] Liu J , Guo Z , Lu S . Baseline physical activity and the risk of severe illness and mortality from COVID-19: a dose–response meta-analysis . Prev Med Rep . 2023 ; 32 : 102130 . doi: 10.1016/j.pmedr.2023.102130 36778629 PMC9905049

[29] Brandenburg JP , Lesser IA , Thomson CJ , Giles LV . Does higher self-reported cardiorespiratory fitness reduce the odds of hospitalization from COVID-19? J Phys Act Health . 2021 ; 18 ( 7 ): 782 - 788 . doi: 10.1123/jpah.2020-0817 33984837

[30] Brawner CA , Ehrman JK , Bole S , et al . Inverse relationship of maximal exercise capacity to hospitalization secondary to coronavirus disease 2019 . Mayo Clin Proc . 2021 ; 96 ( 1 ): 32 - 39 . doi: 10.1016/j.mayocp.2020.10.003 33413833 PMC7547590

[31] Richard SA , Scher AI , Rusiecki J , et al . Decreased self-reported physical fitness following SARS-CoV-2 infection and the impact of vaccine boosters in a cohort study . Open Forum Infect Dis . 2023 ; 10 ( 12 ): ofad579 . doi: 10.1093/ofid/ofad579 38130596 PMC10733205

[32] Jayanama K , Srichatrapimuk S , Thammavaranucupt K , et al . The association between body mass index and severity of coronavirus disease 2019 (COVID-19): a cohort study . PLoS One . 2021 ; 16 ( 2 ): e0247023 . doi: 10.1371/journal.pone.0247023 33592042 PMC7886119

[33] Malik VS , Ravindra K , Attri SV , Bhadada SK , Singh M . Higher body mass index is an important risk factor in COVID-19 patients: a systematic review and meta-analysis . Environ Sci Pollut Res Int . 2020 ; 27 ( 33 ): 42115 - 42123 . doi: 10.1007/s11356-020-10132-4 32710359 PMC7380664

[34] Janvrin ML , Banaag A , Landry T , Vincent C , Koehlmoos TP . BMI changes among US Navy and Marine Corps active-duty service members during the COVID-19 pandemic, 2019–2021 . BMC Public Health . 2024 ; 24 ( 1 ): 2289 . doi: 10.1186/s12889-024-19699-w 39174905 PMC11342622

